# Late‐time window endovascular treatment is associated with neurological improvement: Evidence from the National Stroke Registry Data in China

**DOI:** 10.1111/cns.14572

**Published:** 2024-02-07

**Authors:** Jing Yuan, Z. Kevin Lu, Minghui Li, Jingwen Bai, Long‐De Wang, Renyu Liu, Jing Zhao

**Affiliations:** ^1^ Minhang Hospital & School of Pharmacy Fudan University Shanghai China; ^2^ Department of Clinical Pharmacy and Outcome Sciences University of South Carolina College of Pharmacy Columbia South Carolina USA; ^3^ Department of Clinical Pharmacy and Translational Science University of Tennessee Health Science Center Memphis Tennessee USA; ^4^ The General Office of Stroke Prevention Project Committee, National Health Commission of the People's Republic of China Beijing China; ^5^ Department of Anesthesiology and Critical Care Perelman School of Medicine at the University of Pennsylvania Philadelphia Pennsylvania USA; ^6^ Department of Neurology Minhang Hospital, Fudan University Shanghai China; ^7^ Institute of Healthy Yangtze River Delta, Shanghai Jiao Tong University Shanghai China

**Keywords:** AIS, BOSC, EVT, neurological function

## 1 INTRODUCTION

Stroke is the leading cause of death globally, posing enormous clinical and economic burdens to patients, families, and society.^1^ According to the American Heart Association/American Stroke Association,^2^ endovascular thrombectomy (EVT) is standard care for eligible patients with Acute ischemic stroke (AIS) within 6 h of onset. More recent trials suggested that EVT within the 6–24 h windows or even beyond the 24‐h window may improve functional outcomes.^2^, ^3^, ^4^ However, empirical evidence at different times in a more general population of EVT time window is largely lacking. The objective was to compare the early neurological improvement after EVT in different time windows.

## 2 METHODS

The study employed a retrospective cohort study design by using the Bigdata Observatory Platform for Stroke of China (BOSC), which collects data on incident strokes in 31 out of 34 provinces across mainland China.^5^ We identified adults with a primary diagnosis of AIS who were admitted to the hospital within 7 days of onset and underwent EVT. As described previously,^5^ trained medical staff collected the following data, including sociodemographics, medical history, and lifestyle factors. The National Institutes of Health Stroke Scale (NIHSS) was administered and evaluated upon hospital admission and discharge. The proportion of patients with early neurological improvement (ENI) was defined as a reduction of ≥4 on the NIHSS after EVT^6^; early neurological deterioration (END), which was defined as an increase in NIHSS score, was estimated for each time window. The proportions of patients with ENI/END were compared by using the Chi‐square tests. Because the distribution of NIHSS score was not normally distributed, the medians with upper and lower quartiles were reported and analyzed with the Mann–Whitney *U* test. All analyses were conducted using SAS Version 9.4 (SAS Institute, Inc). Two‐sided *p* < 0.05 was considered statistically significant.

This study was approved by the ethics committees of the Institutional Review Boards of Minhang Hospital at Fudan University. This study followed the Strengthening the Reporting of Observational Studies in Epidemiology (STROBE) reporting guideline.

## 3 RESULTS

Of the 2105 AIS patients who underwent EVT, 1536 (72.97%) presented at hospitals within 6 h, 402 (19.10%) in 6–12 h, 126 (5.99%) in 12–24 h, and 41 (1.95%) over 24 h (Table 1). The mean age is 64.64 years (SD: 11.96), 1426 (67.74%) were aged under 65 years old. The majority of the study sample were men [1426 (67.74%)] and were from Eastern China [640 (30.40%)].

**TABLE 1:** Characteristics of AIS patients who underwent EVT.

Characteristics	All (*n* = 2105)	Time windows			
		≤5.9 h (*n* = 1536)	6.0–11.9 h (*n* = 402)	12.0–23.9 h (*n* = 126)	≥ 24.0 h (*n* = 41)
Age, mean ± SD	64.64 ± 11.96	65.32 ± 12.05	62.50 ± 11.49	63.98 ± 12.39	62.25 ± 9.15
Age (%)					
<65 years	1426 (67.74)	862 (56.12)	180 (44.78)	62 (49.21)	16 (39.02)
65+ years	679 (32.26)	674 (43.88)	222 (55.22)	64 (50.79)	25 (60.98)
Sex (%)					
Male	1426 (67.74)	1005 (65.43)	295 (73.38)	94 (74.60)	32 (78.05)
Female	679 (32.26)	531 (34.57)	107 (26.62)	32 (25.40)	9 (21.95)
Regions^a^ (%)					
Northern China	139 (6.60)	92 (5.99)	28 (6.97)	12 (9.52)	7 (17.07)
Northeast China	136 (6.46)	88 (5.73)	36 (8.96)	12 (9.52)	0
Eastern China	640 (30.40)	501 (32.62)	102 (25.37)	28 (22.22)	9 (21.95)
Central China	535 (25.42)	401 (26.11)	95 (23.63)	30 (23.81)	9 (21.95)
Southern China	333 (15.82)	242 (15.76)	63 (15.67)	24 (19.05)	4 (9.76)
Southwest China	189 (8.98)	134 (8.72)	43 (10.70)	9 (7.14)	3 (7.32)
Northwest China	133 (6.32)	78 (5.08)	35 (8.71)	11 (8.73)	9 (21.95)
Setting (%)					
Rural	1469 (69.79)	1113 (72.46)	250 (62.19)	83 (65.87)	23 (56.10)
Urban	636 (30.21)	423 (27.54)	152 (37.81)	43 (34.13)	18 (43.90)
Medical history (%)					
Stroke	288 (13.68)	200 (13.02)	60 (14.93)	20 (15.87)	8 (19.51)
Cerebral hemorrhage	41 (1.95)	31 (2.02)	8 (1.99)	1 (0.79)	1 (2.44)
Hypertension	1347 (63.99)	979 (63.74)	256 (63.68)	81 (64.29)	31 (75.61)
Diabetes	454 (21.57)	326 (21.22)	76 (18.91)	36 (28.57)	16 (39.02)
Hyperlipidemia	242 (11.50)	161 (10.48)	52 (12.94)	23 (18.25)	6 (14.63)
Onset during night	659 (31.31)	439 (28.58)	148 (36.82)	65 (51.59)	7 (17.07)
Weekend onset	568 (26.98)	423 (27.54)	110 (27.36)	23 (18.25)	12 (29.27)

*Note*: Night time was defined as 6 p.m to 6 a.m.

Abbreviations: AIS, acute ischemic stroke; EVT, endovascular thrombectomy; NIHSS, National Institutes of Health Stroke Scale; SD, standard deviation.

^a^ Northern China includes Beijing, Tianjin, Hebei, Shanxi, and Inner Mongolia; Northeast China includes Liaoning, Jilin, and Heilongjiang; Eastern China includes Shanghai, Jiangsu, Zhejiang, Anhui, Fujian, Jiangxi, and Shandong; Central China includes Henan, Hubei, and Hunan; Southern China includes Guangdong, Guangxi, and Hainan; Southwest China includes Chongqing, Sichuan, Guizhou, Yunnan, and Xizang; Northwest China includes Shaanxi, Gansu, Qinghai, Ningxia, and Xinjiang.

**FIGURE 1:**
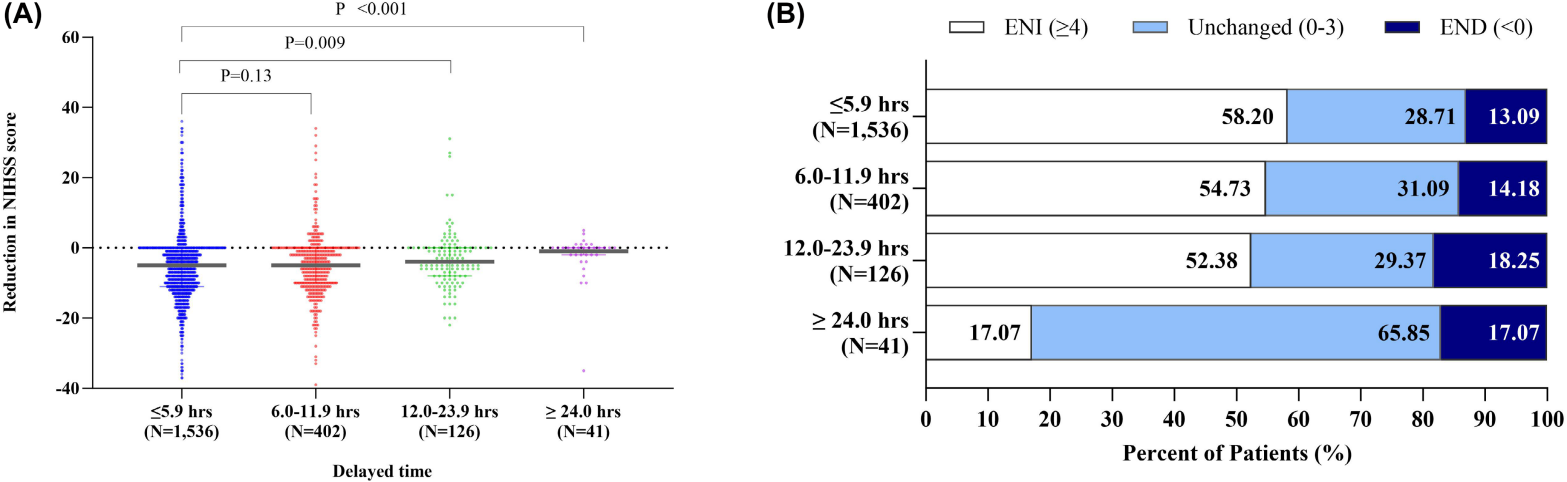
Early neurological function by EVT treatment time window. (A) compares the changes in NIHSS scores by treatment time windows; (B) compares the proportion of patients by change in NIHSS scores and by treatment time windows. CH, Cerebral hemorrhage; END, early neurological deterioration; ENI, early neurological improvement; NIHSS, National Institutes of Health Stroke Scale.

As for the neurological function, the median reduction in NIHSS score (the improvement of neurological function) was 5.00 [interquartile range (IQR): 0–11.00] for patients presenting within 6 h, 5.00 (IQR: 0–10.00) in 6–12 h, 4.00 (IQR: 0–8.00) in 12–24 h, and 1.00 (IQR: 0–2.00) for over 24 h (Figure 1). The rate of ENI in patients over 24‐h time windows was 17.07%, which was only one‐third of those among patients arriving within 6 h (58.20%), in 6–12 h (54.73%), and in 12–24 h (52.38%). The proportion of END in patients who underwent EVT over 24 h was 17.07%, which was similar for those who underwent EVT within 24 h.

## 4 DISCUSSION

In this nationally representative multi‐center analysis of AIS patients, EVT beyond 24 h was associated with early neurological improvement, which is a strong indicator of long‐term functional outcomes.^6^ However, our study reported a much lower proportion of functional improvement, which could be potentially explained by the fact that our findings are based on a more general, representative population in both rural and urban settings across 31 provinces in mainland China, as compared with recently published trials that only focused on specific patients mainly from western countries.^3^, ^4^ Furthermore, the current trials measured the 3‐month functions, which is generally better than early function upon discharge from the hospital. Compared to EVT within 6 h, which is the time window recommended by the current guideline,^2^ the late‐time window of EVT was associated with fewer improvements in neurological functions.

Our study has several limitations. First, patients with missing data were excluded from the analysis, which may lower the generalizability of our findings. Second, some patients and their caregivers may be unable to report the stroke onset time accurately.

## 5 CONCLUSIONS

Our study provides empirical evidence supporting EVT in AIS patients within 24 h of the onset. It also demonstrates the importance of shortening the prehospital delay time but encourages consideration of EVT that is beyond the recommended time window for better neurological improvement in the real world.

## CONFLICT OF INTEREST STATEMENT

The authors declare no conflicts of interest.

## FUNDING INFORMATION

The National Natural Science Foundation of China; CIHR, Grant/Award Number: 81973157, PI: JZ. Natural Science Foundation of Shanghai; CIHR, Grant/Award Number: 17dz2308400, PI: JZ.

## Data Availability

The data that support the findings of this study are available from the corresponding author upon reasonable request.

## References

[1] GBD 2019 Stroke Collaborators . Global, regional, and national burden of stroke and its risk factors, 1990–2019: a systematic analysis for the global burden of disease study 2019. Lancet Neurol. 2021;20(10):795‐820. doi:10.1016/s1474-4422(21)00252-0 34487721 PMC8443449

[2] Powers WJ , Rabinstein AA , Ackerson T , et al. Guidelines for the early Management of Patients with Acute Ischemic Stroke: 2019 update to the 2018 guidelines for the early Management of Acute Ischemic Stroke: a guideline for healthcare professionals from the American Heart Association/American Stroke Association. Stroke. 2019;50(12):e344‐e418. doi:10.1161/str.0000000000000211 31662037

[3] Sarraj A , Kleinig TJ , Hassan AE , et al. Association of Endovascular Thrombectomy vs medical management with functional and safety outcomes in patients treated beyond 24 hours of last known well: the SELECT late study. JAMA Neurol. 2022;80:172‐182. doi:10.1001/jamaneurol.2022.4714 PMC9857518 36574257

[4] Nogueira RG , Jadhav AP , Haussen DC , et al. Thrombectomy 6 to 24 hours after stroke with a mismatch between deficit and infarct. N Engl J Med. 2018;378(1):11‐21. doi:10.1056/NEJMoa1706442 29129157

[5] Yuan J , Lu ZK , Xiong X , et al. Age and geographic disparities in acute ischaemic stroke prehospital delays in China: a cross‐sectional study using national stroke registry data. Lancet Reg Health West Pac. 2023;33:100693. doi:10.1016/j.lanwpc.2023.100693 37181525 PMC10166992

[6] de Campos AM , Carvalho A , Rodrigues M , et al. Ultra‐early improvement after endovascular thrombectomy and long‐term outcome in anterior circulation acute ischemic stroke. J Neurol Sci. 2020;412:116665. doi:10.1016/j.jns.2020.116665 32088468

